# Hepatitis B seroconversion revisited: new insights into the natural history of acute hepatitis B virus (HBV) infection from quantitative and highly sensitive assays and novel biomarkers

**DOI:** 10.1186/s12985-021-01706-w

**Published:** 2021-11-29

**Authors:** Mary C. Kuhns, Vera Holzmayer, Anne L. McNamara, Mark Anderson, Gavin A. Cloherty

**Affiliations:** Infectious Diseases Research, Diagnostics Division, Abbott Laboratories, 100 Abbott Park Road, Abbott Park, IL 60064 USA

**Keywords:** Hepatitis B virus, Acute HBV, Seroconversion, Immunoassays, Biomarkers, HBsAg, HBV pregenomic RNA, HBcrAg, HBV DNA

## Abstract

**Background:**

Hepatitis B virus (HBV) serum markers during typical acute self-limited infection are usually depicted as a composite of traditional HBV markers. The current study updates and expands our knowledge of acute hepatitis B with quantitative molecular and serological data on longitudinal samples from five plasmapheresis donors with acute HBV.

**Methods:**

137 longitudinal samples from five plasmapheresis donors with acute HBV were tested, four with self-limited infection and one who developed persistent infection. Testing included quantitative hepatitis B surface antigen (HBsAg), antibodies to HBV antigens, quantitative HBV e antigen (HBeAg), HBV DNA, quantitative HBV core-related antigen (HBcrAg), the highly sensitive ARCHITECT HBsAg NEXT (HBsAgNx) assay, and a quantitative research assay for HBV pregenomic RNA (pg RNA).

**Results:**

Peak levels of HBV DNA and HBsAg differed by several orders of magnitude among the panels (2.2 × 10^5^–2.7 × 10^9^ IU/ml for HBV DNA and 7.9–1.1 × 10^5^ IU/ml for HBsAg). HBsAg levels peaked an average of 2.8 days after the HBV DNA peak. The overall duration of observed HBsAg positivity was increased by the more sensitive HBsAgNx assay compared to the quantitative assay in four panels. Intermittently detectable low-level HBV DNA was observed after HBsAg loss in three panels. Peak HBeAg levels occurred 2–20 days after the DNA peak and ranged from 1.1 to 4.5 × 10^3^ IU/ml. In four panels with resolution of infection, anti-HBs levels indicating immunity (≥ 10 mIU/ml) were detected 19–317 days after the HBV DNA peak. Maximum HBcrAg concentrations ranged from 1 × 10^5^ to > 6.4 × 10^6^ U/ml and correlated with HBeAg values (R^2^ = 0.9495) and with HBV DNA values (R^2^ = 0.8828). Peak pgRNA values ranged from 1.6 × 10^3^ to 1.4 × 10^8^ U/ml and correlated with HBV DNA (R^2^ = 0.9013).

**Conclusion:**

Traditional and new/novel HBV biomarkers were used to generate molecular and serological profiles for seroconversion panels spanning the early to late phases of acute HBV. Seroconversion profiles were heterogeneous and may be instructive in appreciating the spectrum of acute profiles relative to the typical composite representation.

## Introduction

Viral hepatitis continues to account for significant global disease and high mortality from liver cancer and cirrhosis. In 2019, the World Health Organization estimated that 296 million people were living with chronic hepatitis B virus (HBV) infection worldwide and that there are about 1.5 million new hepatitis B infections each year, despite the availability of a highly effective vaccine [1].

The course of HBV serum markers during a typical acute self-limited HBV infection is usually depicted as a composite of traditional HBV marker data from studies of blood donors [2–4]. Results are displayed as relative concentrations along with the mean lengths of the various phases of acute infection. The order of appearance of serum HBV markers follows a consistent pattern with an eclipse phase preceding the release of virions into the blood, followed by the first detectable levels of HBV DNA, hepatitis B surface antigen (HBsAg), hepatitis B e antigen (HBeAg), and antibodies to HBV core and HBeAg antigens (anti-HBc IgM, total anti-HBc, and anti-HBe). Resolution of infection and recovery is marked by loss of serum HBsAg and HBV DNA and the appearance of antibody to HBsAg (anti-HBs).

The development of persistent infection is indicated by the continued detection of HBsAg for more than six months. Models of the natural history of HBV infection are critical to our understanding of the risk of transmission and reactivation and the sensitivity requirements for assays used in diagnostics and blood screening. In addition, knowledge of the complexities of HBV markers during acute self-limited infection or development of persistent infection is relevant to understanding resolution of chronic hepatitis B in response to anti-viral therapy and in studies of occult HBV infection.

The current study updates and expands our knowledge of acute hepatitis B with data on longitudinal samples from five plasmapheresis donors with acute HBV infection, four with self-limited infection and one with development of persistent infection. We report detailed serological and molecular data on the course of hepatitis B seroconversion using state of the art assays for quantitation of HBsAg and HBeAg, a new highly sensitive HBsAg assay, and novel biomarkers for quantitation of HBV pregenomic RNA (pgRNA) and HBV core related antigen (HBcrAg).

## Materials and methods

### Samples

Hepatitis B seroconversion panels 26022-14518, 13867-3482, 1807-3463, 43527-3453, and 0994–3457 were purchased from North American Biologicals, Inc. (Boca Raton, FL). The panels are series of longitudinal samples from five plasmapheresis donors identified as having acute HBV infection. All were normal source plasma donors, negative for HCV and HIV-1, and anti-viral treatment naïve. All panels are genotype A.

### Serologic and molecular testing

Samples were tested for HBsAg using ARCHITECT HBsAg Qualitative II (analytical sensitivity 0.017–0.022 IU/ml) and with the new qualitative ARCHITECT HBsAg NEXT (HBsAgNx) assay with analytical sensitivity of 0.005 IU/ml (Abbott Diagnostics, Sligo, Ireland) [5, 6]. HBsAgNx is a one-step chemiluminescent microparticle immunoassay with two monoclonal antibodies coated on the microparticles and a goat anti-HBs conjugate. The assay uses 75 ul of specimen (the same as ARCHITECT Qualitative II) with no sample pretreatment. The assay is fully automated and is performed on the Abbott ARCHITECT or Alinity *i* instruments equipped with heat induction probes to eliminate sample carry-over. Performance characteristics of the HBsAgNx assay compared to the Qualitative II assay have been previously reported [5, 6]. Included in those reports were analysis of analytical sensitivity across reagent lots using the 2^nd^ WHO International HBsAg Standard, calculation of clinical sensitivity (with 95% confidence intervals) in acute, chronic, and diagnostic populations, and statistical analysis of specificity in blood donors and diagnostic populations (with 95% confidence intervals, mean S/CO values, and standard deviations for the respective assays). Quantitative HBsAg (qHBsAg), anti-HBs, anti-HBc (total), anti-HBc IgM, quantitative HBeAg, and anti-HBe assays were performed using PRISM, ARCHITECT, or AxSYM assays (Abbott Diagnostics, Abbott Park, IL, USA; Sligo, Ireland; Wiesbaden, Germany). HBV DNA levels were quantitated with Abbott RealTi*m*e HBV (Abbott Molecular, Des Plaines, IL, USA). Late seroconversion samples were tested with multiple replicates to enhance sensitivity for very low levels of HBV DNA [4, 7]. The lower limit of quantitation (LLOQ) for the quantitative ARCHITECT HBsAg assay is 0.05 IU/ml. The LLOQ for the AxSYM and ARCHITECT HBeAg assays are 0.28 and 0.59 IU/ml, respectively. HBcrAg testing was performed with the Lumipulse G HBcrAg assay (Fujirebio, Tokyo, Japan), lower limit of measurement 3 log U/ml. HBV pgRNA was determined using an Abbott research assay with a LLOQ of 1.65 log U/ml (44.7 U/ml) as previously described [8]. Results for samples with detectable pgRNA below the quantitation range are reported as < 1.65 log U/ml.

### Statistics

Correlation analyses were performed using GraphPad Prism 8.0.2.

## Results

**Fig. 1:**
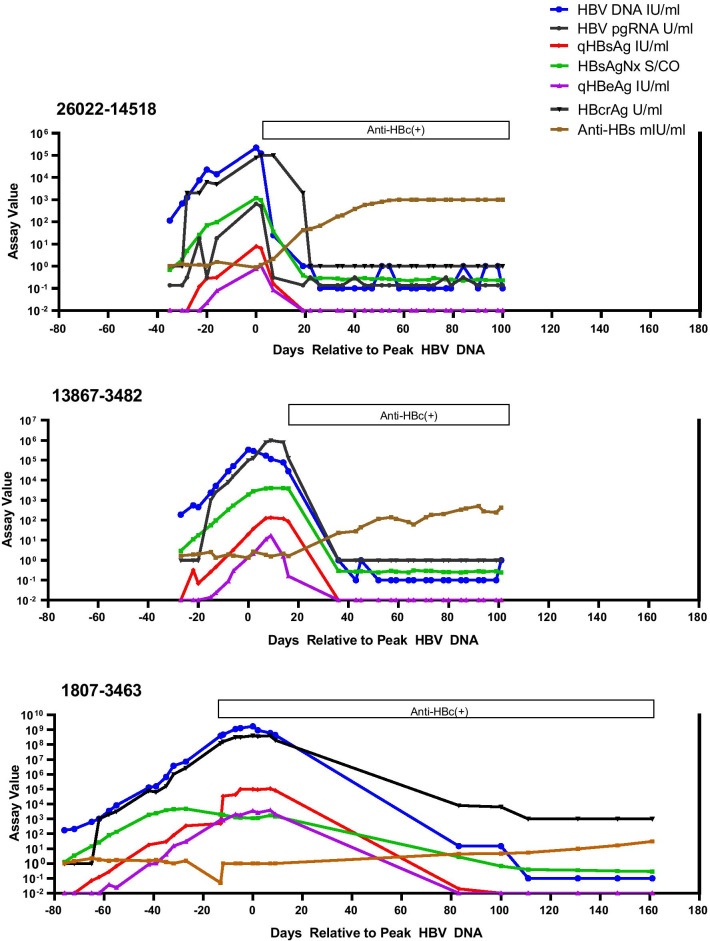
Serological and molecular assay profiles for three seroconversion panels with HBsAg positivity  less than 6 months and resolution of infection with development of anti-HBs ≥ 10 mIU/ml (26022-14518, 13867-3482, 1807-3463). For comparison purposes, all panels are synchronized relative to the peak HBV DNA level (set at Day 0). For graphing purposes, HBV DNA results detected below the LLOQ (10 IU/ml) were assigned the value of 1.0 IU/ml; undetectable HBV DNA was assigned a value of 0.1 IU/ml. HBV pgRNA results (one-tenth scale) detected below the LLOQ (44.7 U/ml) were assigned the value of 2 U/ml; undetectable pgRNA was assigned the value of 1 U/ml. HBcrAg values below the measuring range of the assay were assigned the value of 1 U/ml. Results are considered reactive as follows: Quantitative HBsAg ≥ 0.05 IU/ml, HBsAgNx S/CO ≥ 1.0, Quantitative HBeAg ≥ 0.28 IU/ml, anti-HBs ≥ 10 mIU/ml. HBV pgRNA results for panels 13867-3482 and 1807-3463 were reported elsewhere [8]

**Fig. 2:**
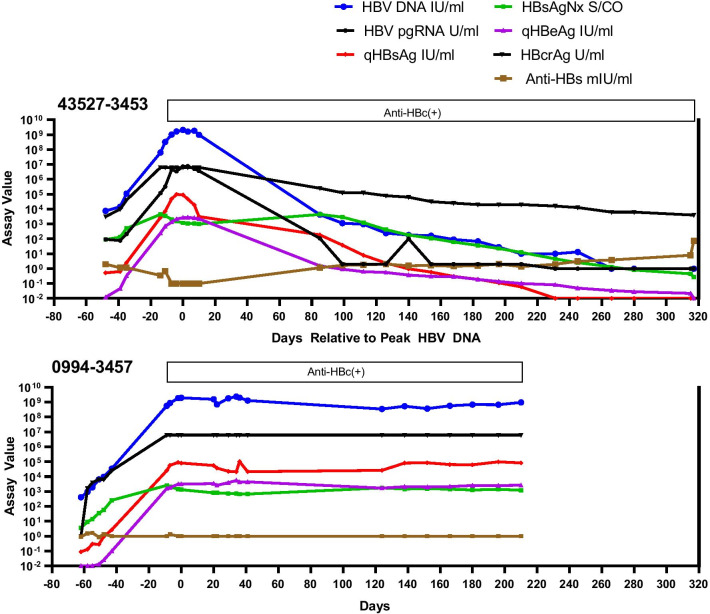
Serological and molecular assay profiles for a panel with prolonged but declining surface antigenemia exceeding 6 months (43527-3453) and a panel with progression to chronicity (0994-3457). For seroconverter 43527-3453, HBsAg became undetectable 266 days after the DNA peak and anti-HBs was detected ≥ 10 mIU/ml at day 317. HBV DNA and HBsAg levels remained high in panel 0994-3457 with no detectable anti-HBe or anti-HBs. For comparison purposes, all panels are synchronized relative to the peak HBV DNA level (set at Day 0). For graphing purposes, HBV DNA results detected below the LLOQ (10 IU/ml) were assigned the value of 1.0 IU/ml; undetectable HBV DNA was assigned a value of 0.1 IU/ml. HBV pgRNA results detected below the LLOQ (44.7 U/ml) were assigned the value of 2 U/ml; undetectable pgRNA was assigned the value of 1 U/ml. HBcrAg values below the measuring range of the assay were assigned the value of 1 U/ml. Results are considered reactive as follows: Quantitative HBsAg ≥ 0.05 IU/ml, HBsAgNx S/CO ≥ 1.0, Quantitative HBeAg ≥ 0.28 IU/ml, anti-HBs ≥ 10 mIU/ml. HBV pgRNA results for panel 0994-3457 were reported elsewhere [8]

A total of 137 samples from five acute HBV infections were studied (Table 1). The number of samples per panel ranged from 23 to 31 with total follow-up ranging from 128 to 365 days. Figures 1 and 2 display the serological and molecular profiles for the five panels. To more readily allow comparisons among the panels, the time of the peak HBV DNA level for each panel was set as Day 0 and all other data are displayed in reference to this point. Figure 1 shows the results for three seroconverters that resolved infection. Figure 2 shows the results for a seroconverter (43527-3453) with prolonged but declining surface antigenemia exceeding six months with eventual loss of detectable HBsAg 266 days after the HBV DNA peak followed by the appearance of anti-HBs. Figure 2 also presents data on a serconverter who progressed to chronicity (0994-3457).

**Table 1:** Characteristics of seroconversion panels in this study

Panel	Total samples	Total days followed	Maximum value				
			HBV DNA (IU/ml)	pgRNA (U/ml)	qHBsAg (IU/ml)	HBeAg (IU/ml)	HBcrAg (U/ml)
26022-14518	31	135	2.2 × 10^5^	1.6 × 10^3^	7.9	1.1	1 × 10^5^
13867-3482	31	128	3.4 × 10^5^	3.5 × 10^3^	134.3	16.8	1 × 10^6^
1807-3463	25	237	1.7 × 10^9^	1 × 10^7^	1.1 × 10^5^	3.8 × 10^3^	3.9 × 10^8^
43527-3453	27	365	2.7 × 10^9^	7.6 × 10^6^	9.7 × 10^4^	2.8 × 10^3^	> 6.4 × 10^6^
0994-3457	23	272	2.4 × 10^9^	1.4 × 10^8^	1.0 × 10^5^	4.5 × 10^3^	> 6.4 × 10^6^

### Quantitation of HBV DNA, HBsAg, and HBeAg

The use of quantitative assays for HBV DNA, HBsAg, and HBeAg provides a detailed picture of the kinetics of these markers during early acute infection. All five panels were positive for low levels of HBV DNA (115–7586 IU/ml) on the first sample obtained (from 27 to 76 days before the HBV DNA peak) (Figs. 1, 2). During the early ramp-up phase, HBV DNA and HBsAg increased in parallel. Parallel increases in HBV DNA and HBsAg have been previously reported using qualitative HBsAg assays [9]. However, the timing and concentration of the HBsAg peak in those earlier studies was not clear due to the limited number of follow-up samples during acute infections and the dynamic range of qualitative assays requiring dilution and titration of HBsAg levels. In the current study, quantitative HBsAg assay results indicated that surface antigen levels peaked an average of 2.8 days after the HBV DNA peak (range 4 days before to 9 days after the DNA peak.) Peak levels of HBV DNA and HBsAg differed by several orders of magnitude among the panels (2.2 × 10^5^–2.7 × 10^9^ IU/ml for HBV DNA and 7.9–1.1 × 10^5^ IU/ml for HBsAg) (Table 1). Interestingly, HBsAg levels declined by one to three logs within 30 days after the HBsAg peak in three of the seroconverters who resolved infection (13867-3482, 26022-14518, and 43527-3453). The interval to the next sample after the HBsAg peak in panel 1807-3463 was 76 days by which time HBsAg was no longer detected by the quantitative HBsAg assay. In contrast, HBsAg levels remained high in the seroconverter who developed persistent infection (0994-3457), declining less than fourfold from the peak value and then increasing again during follow-up. Further studies would be needed to determine the possible predictive value for progression to chronic hepatitis B. Replicate testing of samples from the late seroconversion period revealed that very low levels of HBV DNA (estimated as less than 3 IU/ml) were intermittently detectable in late follow-up samples (one to three months after HBsAg clearance) for panels 43527-3453, 26022-14518, and 13867-3482.

Following detection of HBV DNA and HBsAg, the next traditional HBV antigen marker to be detected is HBeAg with initial detection 6 to 42 days before the DNA peak in four of the panels and concurrent with the DNA peak in one panel (26022-14518). The quantitative HBeAg assay allowed the identification of peak HBeAg levels which occurred 2–20 days after the DNA peak and ranged from 1.1 to 4.5 × 10^3^ IU/ml (Figs. 1, 2, Table 1). Quantitative values for HBeAg and HBV DNA correlated well (R^2^ = 0.7701) consistent with HBeAg as a well-established marker reflecting viral replication.

### High sensitivity HBsAg assay

The highly sensitive HBsAgNx qualitative assay was reactive on the first sample in four of the five panels (equivalent to the time of HBV DNA detection). In one panel (26022–14518), HBsAgNx was reactive on the second sample in the series. For three panels (13867-3482, 26022-14518, and 1807-3463), HBsAgNx (sensitivity 0.005 IU/ml) was reactive 5, 7, and 11 days earlier, respectively, than the quantitative HBsAg assay (sensitivity 0.05 IU/ml). The overall duration of observed HBsAg positivity was increased by the more sensitive HBsAgNx assay compared to the quantitative assay in four panels (Table 2). These results are consistent with previous reports showing that the HBsAgNx assay reduced the early acute HBsAg-negative window by 4.1–4.6 days and lengthened the duration of HBsAg detection in the late acute phase compared to an assay with sensitivity of 0.017–0.022 IU/ml [5, 6, 10]. The prolonged duration of HBsAg positivity in Panel 43527-3453 beyond 6 months fulfilled the criterion for classification as chronic hepatitis B. However, levels of HBV DNA, HBsAg, and HBeAg slowly declined. HBsAg (by HBsAgNx) became undetectable 266 days after the HBV DNA peak and anti-HBs ≥ 10 mIU/ml was detected at day 317.

**Table 2:** Duration of HBsAg positivity (days)

Panel	Quant. ARCHITECT HBsAg	ARCHITECT HBsAg NEXT
26022-14518	30	37
13867-3482	38	43
1807-3463	74*	159
43527-3453	244	314
0994-3457	> 272**	> 272**

^*^Duration may be underestimated due to the long interval to the next available sample

^**^This seroconverter developed persistent infection. The last follow-up sample was 272 days after the HBV DNA peak

### New biomarkers: HBcrAg

The HBcrAg assay detects three antigens expressed from the pre-core/core gene: HBcAg, HBeAg, and a 22 kD pre-core precursor protein [11]. HBcrAg has been reported to reflect the activity of covalently closed circular HBV DNA (cccDNA) in the hepatocyte and to be detectable in the absence of HBV DNA in patients with suppressed viral replication due to treatment with nucleoside analogues. HBcrAg kinetics relative to HBV DNA and HBeAg are shown in Figs. 1 and 2. Maximum HBcrAg concentrations ranged from 1 × 10^5^ to > 6.4 × 10^6^ U/ml (Table 1). HBcrAg values correlated with HBeAg values (R^2^ = 0.9495) and with HBV DNA values (R^2^ = 0.8828). Among 102 HBV DNA positive samples from the five panels, 52.9% were positive for both HBeAg and HBcrAg, 29.4% were HBeAg-negative and HBcrAg-positive, and 17.6% were negative for both HBeAg and HBcrAg. The finding of HBcrAg in the absence of HBeAg likely reflects the multiple proteins targeted by the HBcrAg assay including the HBV core antigen.

### New biomarkers: HBV pgRNA

Recent studies have shown that HBV RNA is detectable in the serum of chronic hepatitis B patients (treated and untreated) as well as in acute infection [12, 13]. The HBV RNA in serum includes primarily full-length encapsidated pgRNA. We have previously reported on the use of a research assay in the quantitation of HBV pgRNA in panels 1807–3463, 13867–3482, and 0994-3457 [8]. In the current study, we expanded the evaluation of pgRNA during acute infection to include panels 43527-3453 and 26022-14518 (Figs. 1, 2). Consistent with our previous results comparing HBV pgRNA and HBV DNA levels (R^2^ values 0.7807–0.9910), HBV pgRNA in the current study consistently paralleled HBV DNA with high correlation between values (R^2^ = 0.9013). HBV pgRNA levels were lower than HBV DNA levels throughout both series of samples with a mean difference of 2.22 log U/ml, similar to the mean difference of 2.25 log U previously reported [8]. Peak values for pgRNA for the five panels ranged from 1.6 × 10^3^ to 1.4 × 10^8^ U/ml (Table 1). The intermittent DNA positivity observed in the late follow-up samples from panel 26022-14518 was also reflected in the intermittent detection of pgRNA during late seroconversion.

### Detection of antibodies

The timing of detection of antibodies to HBV are summarized in Figs. 1 and 2 and Table 3. In four of the five panels, HBeAg levels declined in parallel with HBV DNA followed by seroconversion to anti-HBe positivity. In the four panels with resolution of infection, anti-HBs levels indicating immunity (≥ 10 mIU/ml) were detected 19–317 days after the HBV DNA peak. Neither anti-HBe or anti-HBs were detected in panel 0994-3457, consistent with the development of a persistent infection.

**Table 3:** Timing of antibody detection

Panel	Days relative to HBV DNA peak				
	Anti-HBc IgM First positive	Anti-HBc IgM Last positive	Anti-HBc (total) First positive	Anti-HBe First positive	Anti-HBs ≥ 10 mIU/ml
26022-14518	+ 7	+ 90	+ 2	+ 19	+ 19
13867-3482	+ 36	+ 101	+ 16	+ 14	+ 36
1807-3463	− 5	+ 111	− 13	+ 83	+ 147
43527-3453	+ 7	+ 245	+ 7	+ 317	+ 317
0994-3457	+ 20	+ 180	− 9	NA*	NA*

^*^Not applicable (NA). This seroconverter developed persistent infection. Anti-HBe and anti-HBs were negative at the last follow-up sample 272 days after the HBV DNA peak. Minus sign ( −) indicates time before the HBV DNA peak; plus sign ( +) indicates time after the HBV DNA peak

## Discussion

The current study provides detailed serological and molecular profiles for longitudinal samples from five plasmapheresis donors with acute HBV infection. Included were four seroconversion series with acute resolving infection and one acute series leading to persistent infection. The panels in the current study are uniquely informative in that they encompass the entire seroconversion profile from early acute infection, through the peak levels of HBV DNA and HBsAg, to the decline and loss of detectable DNA and HBsAg and development of anti-HBe and anti-HBs. Although the current report is limited to five panels, it is important to note that HBV seroconversion panels that span the entirety of acute HBV infection are extremely rare. Informative panels require samples obtained during the ascending, peak, and descending portions of the HBV biomarker seroconversion curves through resolution of acute infection and detection of anti-HBs. In addition, sampling intervals should be less than two weeks, preferably one week, with sufficient volume to allow testing of many different biomarkers. A survey of 85 HBV seroconversion panels commercially available over the past decade revealed that only four panels fulfilled the above criteria and none demonstrated progression to chronic infection. The seroconversion profiles of two of the four commercial panels (SCP-HBV-001 from DiaMex, Heidelberg, Germany and PHM935 from SeraCare, Milford MA) were comparable to panel 43527-3453 in the current study, i.e., prolonged declining surface antigenemia. The profiles for the other two commercial panels (6281 from Zeptometrix, Franklin, MA and SCP-HBV-002 from DiaMex) were similar to seroconverter 13867-3482 from the current study.

The current study is distinctive in using quantitative assays for HBV DNA, HBsAg, and HBeAg; a new highly sensitive HBsAg assay; and quantitative assays for two new HBV biomarkers, HBV pgRNA and HBcrAg. Quantitative assays for HBsAg and HBeAg are now widely available. These assays are standardized against WHO International Reference Standards; results are expressed as IU/ml facilitating comparison of data across studies. Previously, depictions of HBV seroconversion relied on results from qualitative assays, which limited the accuracy of determining the timing and concentrations of peak antigen levels. In the current study, the quantitative assays showed that the peak surface antigenemia occurred from 4 days before to 9 days after the HBV DNA peak (Figs. 1, 2). HBeAg levels consistently peaked after HBV DNA ranging from 2 to 20 days later. A wide range of maximum HBV DNA and HBsAg levels were observed in this study. Panels 26022-14518 and 13867-3482 with relatively short durations of infection had the lowest peak levels of HBV DNA and HBsAg (2.2 × 10^5^–3.4 × 10^5^ HBV DNA IU/ml and 7.9–134.3 HBsAg IU/ml) (Table 1). HBV DNA and HBsAg levels in panels 1807–3463, 43527-3453, and 0994-3457 were orders of magnitude higher (1.7 × 10^9^–2.7 × 10^9^ HBV DNA IU/ml and 9.7 × 10^4^–1.1 × 10^5^ HBsAg IU/ml). Maximum HBeAg levels also demonstrated orders of magnitude differences among the panels (Table 1). For comparison, mean HBV DNA levels of 10^7^–10^8^ IU/ml and mean HBsAg levels of 2.3 × 10^4^–9 × 10^4^ IU/ml have been reported for patients with HBeAg positive chronic infection [14].

Previous studies have estimated the mean duration of HBsAg positivity using an assay with 0.02 IU/ml sensitivity. One study provided an estimate of 63 days based on the numbers of HBV nucleic acid test (NAT) positive blood donor samples with detectable HBsAg [4]. Another study reported a mean of 92.7 days based on evaluation of 10 seroconversion panels [15]. The duration of HBsAg positivity is dependent on the sensitivity of the HBsAg assay used. The HBsAgNx assay with 0.005 IU/ml sensitivity has been shown to reduce the early acute HBsAg negative window by 4.1–4.6 days and lengthen duration of HBsAg detection in the late acute phase in comparison with an assay with 0.02 IU/ml sensitivity [5, 6, 10]. In the current study, series 26022-14518 and 13867-3482 had relatively short infections based on the duration of HBsAgNx positivity (37 and 43 days, respectively) while the duration was longer in panel 1807-3463 (159 days) (Table 2).

Interestingly, only two samples were HBeAg positive in the 26022-14518 panel: at days 0 and 2 relative to the DNA peak. The possibility of such a short period of HBeAg positivity should be considered when interpreting patient test results when evaluation of HBV infection depends on only one sample or limited follow-up. HBcrAg results were positive for both of the HBeAg positive samples and for samples immediately preceding and following.

In contrast to the panels with short or intermediate durations of infection, a prolonged course was observed in panel 43527-3453 where HBsAgNx was positive for 314 days before becoming negative 266 days after the HBV DNA peak; anti-HBs was detected at day 317 (Fig. 2, Table 3). By the criterion for classification of chronic infection (HBsAg positivity exceeding six months), this panel could represent a chronic infection which spontaneously resolved. Alternatively, the observation that HBV DNA was still intermittently detectable at very low levels after the loss of HBsAg suggests that this could represent an early chronic infection that became occult (anti-HBc positive, low level HBV DNA positive, HBsAg negative, with low level anti-HBs). The continued high levels of HBV DNA and HBsAg with no detectable anti-HBs in panel 0994-3457 clearly indicate the development of persistent infection.

Although it is common for HBV DNA to be detectable for about 10 days after HBsAg clearance (the so-called post-HBsAg or second window period), intermittent detection of very low levels of HBV DNA in serum years after clearance of serum HBsAg and recovery from self-limited infection has been reported [2]. The existence of HBV DNA PCR positive, HBsAg negative sera from cases of resolving HBV infection and the continued presence of HBV cccDNA and HBV RNA in liver years after HBsAg clearance has been documented [16, 17]. Using the strategy of replicate testing to enhance detection, we found that three of the panels in this study (43527-3453, 26022-14518, and 13867-3482) had intermittent low-level HBV DNA (estimated at ≤ 3 IU/ml) in late follow-up samples. In panels 26022-14518 and 13867-3482, samples with very low DNA levels were observed in the presence of relatively low anti-HBs (25–312 mIU/ml in 13867–3482) and in samples with high anti-HBs (> 1000 mIU/ml in 26022-14518).

Serum HBV pgRNA has emerged as a new biomarker reflecting the levels and activity of intrahepatic HBV covalently closed circular DNA (cccDNA) [13]. HBV pgRNA can be released into the serum within enveloped virions. A dual-target RT-PCR assay has been developed to quantitate HBV pgRNA [8]. HBV pgRNA results from the current study augment and support our previous report showing similar kinetics for HBV DNA and HBV pgRNA during seroconversion, including panel 0994-3457 which progressed to persistent infection [8]. Concentrations of pgRNA were an average of 2.2 logs lower than HBV DNA in the present study, as was found previously. To the authors’ knowledge, these are the only studies reporting the kinetics of HBV pgRNA for longitudinal samples from acute HBV infections. Interestingly, panel 26022-14518 in which we observed intermittently detectable low-level HBV DNA after HBsAg clearance, also had low levels of HBV pgRNA during the same time period, suggesting continued transcriptional activity of HBV cccDNA in the liver.

HBcrAg is comprised of three antigens expressed from the pre-core/core gene: HBcAg, HBeAg, and a 22 kD pre-core precursor protein [11]. The three proteins overlap, sharing an identical 149 amino acid sequence. HBcrAg can be detected in virions containing HBV DNA, DNA-negative particles, and possibly in pgRNA-containing particles. HBeAg is the predominant component detected by the HBcrAg assay in HBeAg positive sera. HBcrAg has been shown to correlate with HBV cccDNA and has been extensively studied relative to the phases of chronic hepatitis B infection and in monitoring antiviral therapies [13, 18]. In the current study of acute hepatitis B, HBcrAg kinetics were similar to HBeAg and HBV DNA and HBcrAg correlated with both markers. Thirty HBV DNA-positive HBeAg-negative samples were positive by the HBcrAg assay, likely due to the multiple proteins detected by the HBcrAg assay. The lack of an international standard and the inability to distinguish among the different HBcrAg components limits further analysis.

An interesting observation from this study is the heterogeneity in the duration of HBsAg and HBV DNA positivity. Although few similar panels are offered by commercial vendors, the seroconversion series in the current study nevertheless are representative of acute HBV infection as supported by comparison with four other commercial panels (discussed above) and by the kinetics of HBV DNA and HBsAg during the ramp-up phase. Biswas et al. reported a mean HBV viral load doubling time of 2.56 days with HBsAg increasing in parallel with HBV DNA during the ramp-up phase for 23 seroconversion panels [9]. For the five panels in the current study, viral load doubling times ranged from 2.0 to 2.7 days with HBsAg increasing in parallel with HBV DNA (Figs. 1, 2) [19]. Thus, the observed differences in duration of surface antigenemia may reflect the natural variability among individuals with acute HBV and may be instructive in appreciating the spectrum of acute profiles relative to the typical composite seroconversion profile and in the interpretation of patient results.

## Conclusions

This study synthesized data on traditional and new/novel HBV biomarkers to generate detailed molecular and serological profiles for five seroconversion panels spanning the early to late phases of acute HBV infection. State-of-the-art quantitative assays for HBV DNA, HBsAg, HBeAg, HBV pgRNA, and HBcrAg along with a new highly sensitive HBsAg assay provided an updated perspective on the natural history of acute HBV infection.

The updated profiles presented herein may be helpful in appreciating the spectrum of acute infection profiles relative to the typical composite seroconversion curves. The results of this study may also be useful for comparisons with the course of HBV markers during anti-viral therapy induced resolution of chronic infection and in understanding the underlying mechanisms of antiviral agents in development.

## Data Availability

The data sets generated and analyzed during this study are included in the published article or are not publicly available, but are available from the corresponding author on reasonable request.

## Abbreviations

HBsAg: Hepatitis B surface antigen
HBV DNA: Hepatitis B virus DNA
HBeAg: Hepatitis B e antigen
HBcAg: Hepatitis B core antigen
anti-HBe: Antibody to hepatitis B e antigen
anti-HBc and anti-HBc IgM: Antibody to hepatitis B core antigen
anti-HBs: Antibody to hepatitis B surface antigen
HBV pgRNA: Hepatitis B virus pregenomic RNA
HBcrAg: Hepatitis B core related antigen
cccDNA: Covalently closed circular DNA
NAT: Nucleic acid test
